# Causal evidence that intrinsic beta-frequency is relevant for enhanced signal propagation in the motor system as shown through rhythmic TMS

**DOI:** 10.1016/j.neuroimage.2015.11.020

**Published:** 2016-02-01

**Authors:** Vincenzo Romei, Markus Bauer, Joseph L. Brooks, Marcos Economides, Will Penny, Gregor Thut, Jon Driver, Sven Bestmann

**Affiliations:** a Wellcome Trust Centre for Neuroimaging at UCL, Institute of Neurology, University College London, London, UK; b UCL Institute of Cognitive Neuroscience, University College London, London, UK; c Department of Psychology, Centre for Brain Science, University of Essex, Colchester, UK; d School of Psychology, The University of Nottingham, Nottingham, UK; e School of Psychology, University of Kent, Canterbury, UK; f Centre for Cognitive Neuroimaging, Institute of Neuroscience and Psychology, University of Glasgow, Glasgow, UK; g Sobell Department of Motor Neuroscience and Movement Disorders, UCL Institute of Neurology, University College London, London, UK

## Abstract

Correlative evidence provides support for the idea that brain oscillations underpin neural computations. Recent work using rhythmic stimulation techniques in humans provide causal evidence but the interactions of these external signals with intrinsic rhythmicity remain unclear. Here, we show that sensorimotor cortex follows externally applied rhythmic TMS (rTMS) stimulation in the beta-band but that the elicited responses are strongest at the intrinsic individual beta peak frequency. While these entrainment effects are of short duration, even subthreshold rTMS pulses propagate through the network and elicit significant cortico-spinal coupling, particularly when stimulated at the individual beta-frequency.

Our results show that externally enforced rhythmicity interacts with intrinsic brain rhythms such that the individual peak frequency determines the effect of rTMS. The observed downstream spinal effect at the resonance frequency provides evidence for the causal role of brain rhythms for signal propagation.

## Highlights

• Subthreshold rhythmic TMS at beta-frequency entrains the motor cortex at rest.
• TMS entrainment in the motor cortex is maximal at the individual beta-frequency.
• Subthreshold TMS induces cortico-spinal coupling at this resonance frequency.
• Cortico-spinal network acts like a beta-band-pass filter even at low muscle tone.

## 1 Introduction

Rhythmic brain activity has been proposed to structure neural information processing (Lakatos et al., 2005, Lakatos et al., 2008) and is known to be modulated by cognitive demands during behavioural and perceptual tasks (Jensen et al., 2007, Womelsdorf and Fries, 2007). Rhythms of different frequencies are thought to play distinct roles in neural processing, even if no direct mapping to cognitive processes exists. For example, alpha-oscillations are thought to be instrumental for filtering of distractive information (Thut et al., 2011a, Romei et al., 2010, Romei et al., 2012, Bauer et al., 2012) and seem generally associated with inhibited cortical states (e.g., Jensen and Mazaheri, 2010, Pfurtscheller et al., 1996, Klimesch et al., 2007). Theta-oscillations may serve as a clock that provides contextual information for neuronal signals (Skaggs et al., 1996, Lee et al., 2005) and may facilitate large-scale integration of information processing (Romei et al., 2011). Beta-oscillations, although more ubiquitously found throughout the brain but most prominently observed in the motor system (e.g., Jensen et al., 2005, Bauer et al., 2012), have been implicated with the cortical control of motor output (Brown, 2000, Engel and Fries, 2010, Brittain et al., 2014). This view is bolstered by the fact that abnormal beta activity is seen in pathological states such as Parkinson’s disease (Little and Brown, 2014).

In addition to this relatively general classification into different bands, recent work has highlighted the importance of individual frequency peaks within these bands. These appear to be informative of physiological phenotype (Muthukumaraswamy et al., 2010), which are determined by genetic factors (van Pelt et al., 2012) and, crucially, are predictive for behavior (Edden et al., 2009, Cecere et al., 2015). Nevertheless, most of this evidence is of correlative nature. Recent studies using interventional neurostimulation approaches in humans have begun to provide evidence for a causal role of these frequency bands using rhythmic TMS (Thut et al., 2011a, Thut et al., 2011b, Romei et al., 2010, Romei et al., 2011, Romei et al., 2012, Sauseng et al., 2009, Klimesch et al., 2003, Chanes et al., 2013, Jaegle and Ro, 2014, Ruzzoli and Soto-Faraco, 2014, Hanslmayr et al., 2014) or transcranial alternating current stimulation (tACS) (Pogosyan et al., 2009, Feurra et al., 2011, Joundi et al., 2012, Neuling et al., 2012, Brittain et al., 2013, Strüber et al., 2014, Helfrich et al., 2014, Cecere et al., 2015).

However, it remains unclear how such rhythmic extrinsic stimulation interacts with intrinsically generated oscillations. More specifically, it remains unknown (a) what the nature of the interaction of extrinsic rhythmicity and the intrinsic rhythm is and (b) how inter-individual differences in intrinsic frequencies may affect the outcome of direct brain stimulation. Because of prior evidence for a causal role of beta-oscillations in the motor system and the operational advantages of stimulating a well-described anatomical region at equivalent TMS intensities (both validated by motor-evoked potentials measured by EMG), we capitalized on sensorimotor beta-oscillations as a model system to address these questions. We first estimated participant’s individual beta-frequency (IBF) so that we could apply subthreshold repetitive TMS at precisely this frequency over motor cortex, in addition to randomly interleaved stimulation trials at four surrounding frequencies (spaced ± 3 and ± 6 Hz from the IBF). Based on previous findings, we expected that the extrinsic stimulation frequency should lead to rhythmic brain activity at any of the active rTMS frequencies (Thut et al., 2011b). More specifically, we hypothesized (i) that stimulation at the IBF should result in a stronger entrainment effect and (ii) a graded effect of entrainment as a function of the distance from the IBF.

## 2 Materials and methods

### 2.1 Participants

Thirteen healthy participants were recruited to participate in the study and received financial compensation for their time spent. Three of them had to be excluded due to excessive electrical artifacts in the EEG signal. The remaining 10 participants had a mean age of 27.2 (range 21–36; 4 females) and were right handed by self-report. For EMG analysis, we had to exclude one more participant because stimulation during the main experiment often evoked MEPs. All signed the written consent form and had no contraindications to TMS. The study was approved by the local ethics committee and was conducted in accordance with the latest TMS safety guidelines (Rossi et al., 2009).

### 2.2 Individual motor beta-frequency peak identification

**Fig. 1:**
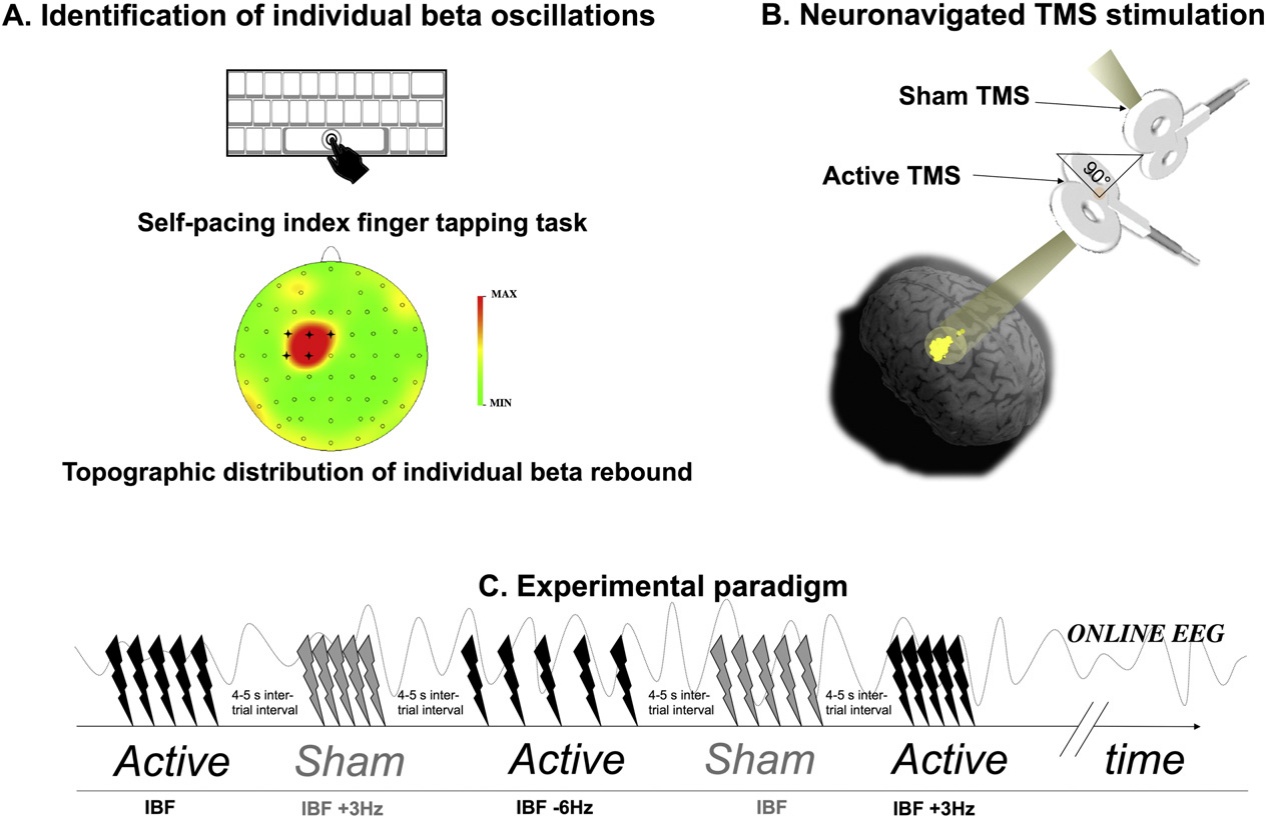
(A) Identification of individual beta-oscillations. A self-pacing right index finger tapping task has been employed to obtain a beta-rebound measure, the topographical distribution of which was localized over left M1 sensors. (B) Localization of motor hand area and subsequent neuronavigated TMS stimulation. Anatomical localization to the omega-shaped knob on the precentral gyrus served to optimize functional localization of the left motor hand area via online observation of motor-evoked potentials during neuronavigated TMS stimulation. To keep track of effective stimulation site over time, neuronavigation was used online to TMS stimulation throughout the experimental session. Each yellow dot shown on the brain surface represents the spatial location of a single TMS trial, from one representative participant. (C) Experimental paradigm. TMS was applied in short bursts of 5 pulses over the motor hand area of left M1. Five frequencies were used in random event-related order. These consisted of a tailored individual beta peak (IBF) as identified above, plus 4 flanking frequencies—IBF ± 3 Hz, IBF ± 6 Hz. TMS intensity was set to 90% of the active motor threshold, so did not evoke any motor-evoked potential in the ongoing EMG signal. Blocks consisted of alternating active/sham stimulations via a control design.

We used a simple self-paced right index finger tapping task on the keyboard’s spacebar of the computer, at a pace of approximately 0.25 Hz, with one hundred repetitions. The beta-rebound following the self-paced finger movement was estimated (prior to the TMS intervention and therefore separately from the reminder of the EEG analyses) by a frequency analysis on artifact-free epochs between 300 and 800 ms post movement using BrainVisionAnalyzer, with a 0 Hanning window allowing a nominal resolution of 0.125 Hz. The localization of the individual beta peak was identified over left M1 sensors (typically electrode C3) with average individual beta-frequency over all participants being 17.54 ± 0.84 Hz (± SEM) (range 14.7–22.6 Hz) (see Fig. 1A).

### 2.3 TMS paradigm

#### 2.3.1 Localization of motor hand area and active motor threshold (aMT) evaluation

Following Yousry et al. (1997), the coil was first positioned via neuronavigation (Visor TMS Neuronavigation System, ANT Advanced Neuro Technology, Netherland) to the anatomical landmark for the left motor hand area identified as an omega-shaped knob on the precentral gyrus on each participant’s individual MRI. Subsequently, a functional localization of the same region was determined via online observation of motor-evoked potentials (MEPs) and index finger twitch. Finally, once the “hot spot” corresponding to the optimal coil position able to elicit MEP of maximal amplitude was identified, an active motor threshold was defined as follows: participants were required to slightly contract their index finger by opposing to the thumb while single TMS pulses were delivered over the “hot spot.” Active motor threshold (aMT) was then defined as the minimum intensity of stimulation able to elicit an MEP of at least 50 μV in at least 3 out of 6 consecutive stimulations. The aMT was on average 46.56% ± 4.93% (SEM) of maximal stimulator output. Intensity of stimulation was set to 90% of aMT (41.9% ± 4.44% (SEM)).

### 2.4 Experimental design

TMS was applied at rest while participants were seated in a comfortable reclining chair. Short bursts of 5 pulses were applied over the motor hand area of left M1. Five frequencies were applied in random event-related order. These consisted of a tailored individual beta peak (IBF) [see above], with 4 flanking frequencies—IBF ± 3 Hz, IBF ± 6 Hz within the beta range. Blocks consisted of alternating active/sham stimulations via a control design (see Fig. 1C), with the active and sham 70 mm figure-of-eight coils being connected to two separate Magstim^2^ stimulators. The active TMS coil was oriented 45° from the midline and the handle posteriorly oriented and connected to one of the two rapid^2^ Magstim biphasic systems. Active coil position was determined via online neuronavigation (see above) while the sham coil was positioned on top of and perpendicular to the active coil (Fig. 1B). This sham condition was also introduced to control for entrainment through rhythmic acoustic stimulation (cf. Romei et al., 2010, Romei et al., 2011, Romei et al., 2012, Thut et al., 2011a, Mathewson et al., 2010, de Graaf et al., 2013).

### 2.5 EEG recording

Using a TMS compatible EEG equipment (ASA-LAB, ANT Advanced Neuro Technology, Netherland), EEG was continuously acquired from 63 channels plus a ground electrode placed at position AFz (WaveGuard EEG Cap, ANT Advanced Neuro Technology, Netherland) using an average reference montage. The signal was digitized at a sampling rate of 2 kHz and skin/electrode impedance was kept below 5 kΩ throughout the experiment.

### 2.6 EMG recording

Electromyographic (EMG) activity was recorded by means of two disposable surface Ag/AgCl auto-adhesive hydrogel clip electrodes placed over the right first dorsal interosseous muscle (FDI) in a belly-tendon montage. Electrodes were connected to EMG-dedicated bipolar channels integrated in the ANT EEG system. The ground electrode corresponded to the EEG ground electrode placed over the scalp. The recording sites over the index finger were cleaned thoroughly with alcohol pads in order to keep electrical impedance below 10kΩ. The EMG raw signal was amplified (ANT Advanced Neuro Technology, Netherland), band-pass filtered (2 Hz to 500 Hz), and digitized at a sampling rate of 2 kHz.

### 2.7 EEG and EMG analysis

Analysis was performed using the Fieldtrip software package (Oostenveld et al., 2011), custom-written MATLAB code, and Brain Vision Analyzer 1 (Brain Products).

#### 2.7.1 Pre-processing and artifact removal

Pre-processing epochs were of 3 s duration (− 1.5; + 1.5 s from TMS train onset), and the artifact removal comprised the following steps: first, the line noise artifact was removed by fitting and subtracting a 50 Hz complex exponential function from the entire epoch around the TMS train. Therefore, power and phase of the 50 Hz line noise was estimated in a 300 ms window ending 200 ms before TMS onset and a 300 ms window starting approximately 200 ms after TMS offset (hence sparing the period containing TMS artifacts). This was achieved by multiplication of the hanning-tapered time series with a complex exponential at the given frequency, as implemented in ft_freqanalysis_convol.m in Fieldtrip. The precise window onset of the latter window was calculated such that it reflected an integer multiple of the 50 Hz cycle. The resulting complex exponential (estimated from pre- and post-TMS window) was then subtracted from the entire epoch (for a similar approach, cf. Thut et al., 2011a). Second, the electrical artifacts associated with the TMS pulses consisted of transient high voltage peaks. These artifacts typically lasted 3 to 8 ms, as reported also by others (e.g., Thut et al., 2011a, Veniero et al., 2009). We removed and replaced these periods by a linear interpolation for a conservative 12 ms window around each TMS pulse (3 ms before and 9 ms after TMS onset), thereby taking out the artifact directly induced by the TMS pulse on the EEG system. Third, all trials (irrespective of condition) were visualized using the fieldtrip function ft_rejectvisual.m and trials with excessive noise were “manually” eliminated. Fourth, to suppress residual artifacts from scalp electrodes such as TMS recharge artifacts (see Veniero et al., 2009), a principal component analysis (PCA, an eigenvector decomposition of the signal resulting from the rotation into an orthogonal vector space with principal components successively extracting maximal variance) was calculated over the samples that were free of the immediate TMS artifact on the recorded signals and from which electrical line noise (50 Hz) had already been suppressed. The leading six PCA-component topographies (or principal components, i.e., the eigenvectors in channel-space) were then graphically displayed and non-dipolar sources or eye-blink topographies were removed. We used PCA due the feature that the Eigenvectors are sorted according to variance extraction and, hence, are defined by an objective criterion. For other examples of using this or closely related techniques, see for instance Wallstrom et al. (2004), Litvak et al. (2007), Rosa et al. (2010). We note that PCA, while having some procedural advantages as laid out above, is known to be a conservative procedure with the risk of extracting cranial signals along with the artifact (see Jung et al., 2000, Litvak et al., 2007), although see also Wallstrom et al. (2004) for other advantages of PCA. However, in a study like this, we considered a more conservative procedure to be favorable, even if at the risk of false rejections (see discussion). Finally, in the last step, individual trials with higher remaining noise levels (often trials with non-stationary 50 Hz noise) or individual channels were manually rejected using the function “ft_rejectvisual” in FieldTrip, a semi-automatical rejection tool. The bipolar EMG channel was included in this entire procedure, except for the PCA removal.

Artifact corrected trials were convolved with complex exponentials (Eq. (1), tapered with a Hanning window, length 0.4 s) in steps of 25 ms in the time and 0.5 Hz in the frequency domain to obtain complex Fourier spectra for frequencies from 5 to 30 Hz and from − 1 to 1 s around TMS (or sham) stimulus onset.

#### 2.7.2 EEG time frequency analysis

(1) $$S\left(w\right)=x\left(t\right)*A\left(w\right)*e^{-i\omega t+\varphi }$$

In order to increase the signal to noise ratio of the measured cortical responses, further analyses were conducted on a virtual channel. To this end, the electrode with the maximal power change during the post-TMS interval (0–0.2 s) relative to baseline (− 0.4 to − 0.2) served as a reference electrode, and the cross-spectral density was calculated of all scalp electrodes to this reference electrode. The estimated cross-spectral density of all electrodes along the real axis (to the reference electrode) was then calculated for the weighting coefficients, following a similar approach taken by Guderian and Düzel, 2005. The rationale behind this procedure was the following: a cortical dipole induces currents in scalp electrodes of opposite polarity (reflecting current directions from source to sink) that are either in zero-phase synchrony or shifted by 180° and therefore this signal will be located on the real axis of the complex cross-spectral density matrix with respect to the electrode that contains the maximal signal from this dipole. The same frequency analysis was then repeated for the time series obtained from the virtual channel, and the same analyses as performed for the sensor level were then conducted on these data.

Specifically, we calculated the power spectral density (or auto-spectrum, see Eq. (2)) in the specified time and frequency range of each condition to the test for the amplitude of the TMS-induced effects (*S*_i_ represents the complex Fourier coefficients at angular frequency *w* for trial *i*):

(2) $${PSD}_{i}\left(w\right)=\frac{S_{i}\left(w\right)*S_{i}\left(w\right)'}{L}$$

To investigate specifically how TMS pulses entrain the brain signals, we also calculated the phase-locking values of cortical potentials to the TMS train (see Eq. (3)). To this end, we used the trigger channel giving the specific timing of the TMS pulses (e.g., as channel *x*) and multiplied its complex Fourier spectra with the complex complement of those of the EEG/virtual channel *y*. This cross-spectral density between *x* and *y* was then normalized for each trial on the respective autospectra to obtain complex cross-spectra on the unit circle simply reflecting the phase difference between *x* and *y*. Averaging these coefficients gives a measure of the inverse variance of the phase difference, hence the phase-consistency (or phase-locking) between the TMS and the EEG signal:

(3) $${PLV}_{xy}\left(w\right)=\;\sum_{i}^{n\;\mathrm{trials}}\frac{Sx\left(w\right)*Sy'\left(w\right)}{(\sqrt{Sx\left(w\right)*Sx'\left(w\right)}*\;\sqrt{Sy\left(w\right)*Sy'\left(w\right))}}$$

We note here that while our artifact treatment (for each pulse and hence at the given frequency) will by itself lead to some degree of phase locking (and therefore the mere existence of such phase locking is of less interest), the key issues are the differences between conditions (the different frequencies as a function of distance from the IBF and the active vs. sham TMS condition). To finally test for “remote” effects of the TMS pulses, we further investigated cortico-muscular coherence, a standard measure to investigate interactions between motor cortex and spinal neurons (Schoffelen et al., 2005). This is calculated (see Eq. (4)) in the same way as the phase-locking value, but now channel *x* represents the EMG channel, and the cross-spectra are not normalized for the individual trials but the sum of the cross-spectra is normalized to the sum of the autospectra. This measure is thus influenced by both phase-consistency and amplitude covariations (as in cross-correlation which is the Fourier transform of coherence).

(4) $$Coh_{xy}\left(w\right)=\frac{\sum_{i}^{n\;\mathrm{trial}}Sx\left(w\right)*Sy'\left(w\right)}{\sum_{i}^{n\;\mathrm{trials}}\sqrt{Sx\left(w\right)*Sx^{\prime }\left(w\right)}*\sum_{i}^{n\;\mathrm{trials}}\sqrt{Sy\left(w\right)*Sy'\left(w\right)}}$$

#### 2.7.3 EMG analysis

The time frequency analysis for the EMG channel followed the same procedure as described for the EEG (apart from EMG being used for coherence analysis anyhow) and was performed for both the raw (bipolarly recorded) EMG signal as well as the rectified EMG signal. Both results delivered qualitatively the same results and so we followed a recommendation by McClelland et al., 2012 and used the non-rectified signal.

In order to investigate whether any of the observed effects might be due to EMG responses evoked by the rTMS-protocol, we investigated the EMG for presence of motor-evoked potentials (MEPs) as well as any signs of TMS-evoked activations or artifacts. Typically, motor threshold is defined as an EMG deflection of approximately 50 mV, as we determined in our motor threshold estimation procedure. In other words, the motor threshold is generally defined as the lowest TMS intensity able to induce MEPs of 50 mV (peak-to-peak amplitude) in that given muscle in at least the 50% of the trials (Rossini et al., 1994), when measured with surface electrodes. To investigate randomly occurring suprathreshold activations, we adopted a more liberal criterion and considered an evoked motor response as any peak-to-peak difference of at least 20 mV (i.e., less than a half the minimum amplitude defined to be at threshold level) within a time window from 18 to 27 ms after each individual TMS pulse. This time window corresponds to the likely time of MEP occurrence and reflects the possible range of conduction times from the stimulation site to the contralateral muscles. Hence, the EMG time series of all trials were searched according to this criterion and threshold passes counted. We also calculated the root-mean square (RMS) in the EMG traces during this 18–27 ms period. In one participant, the EMG traces were particularly noisy during the main experiment and this participant was therefore excluded from all subsequent analyses that included the EMG trace (in particular also the cortico-spinal coherence).

#### 2.7.4 Statistical analysis

A repeated-measure analysis of variance (ANOVA) was performed with the within factors stimulation type (active vs. sham) and stimulation frequency (IBF vs. surrounding frequencies). Our hypothesis supports the notion that all conditions tested here can lead to rhythmic brain activity (cf. Thut et al., 2011b). However, the key question was whether stimulation at IBF would lead to stronger entrainment than other frequencies. In order to directly test this assumption, maximize statistical power, and reduce the number of comparisons, we contrasted the IBF against the average of all surrounding frequencies, thereby eliminating the influence of factors such as a monotonic dependency of the induced brain waves by stimulation frequency.

Finally, inspection for presence/absence of MEPs in the EMG was carried out.

## 1.3 Results

### 3.1 TMS effects on motor cortical beta-power

**Fig. 2:**
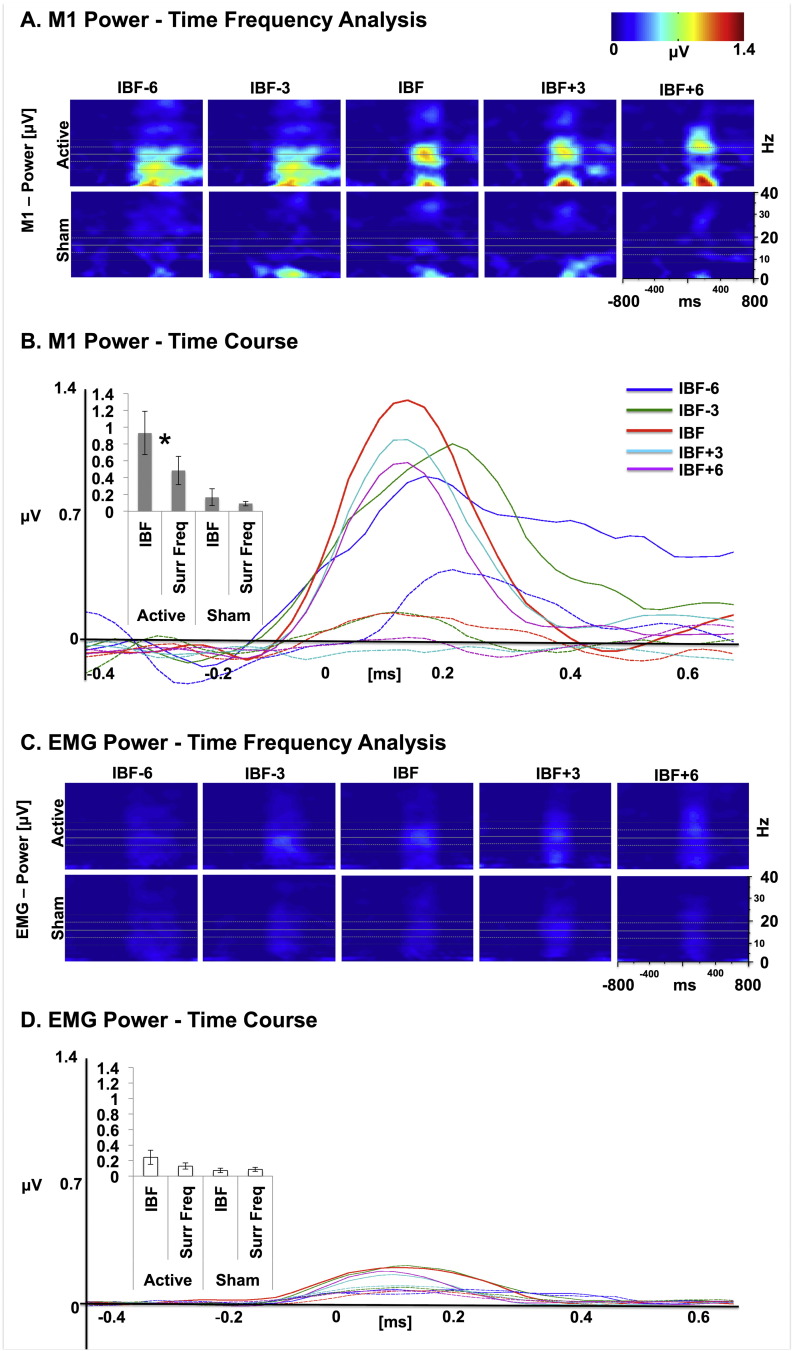
(A) M1 power–time frequency analysis. Entrainment effects for M1 power as a function of frequency of stimulation (individual beta-frequency [IBF], central plot and surrounding frequencies: IBF ± 3 Hz and IBF ± 6 Hz) and condition (active stimulation: upper row; sham stimulation: lower raw). The set of horizontal lines in scatterplots represent the average IBF (central line), average IBF ± 3 Hz (middle lower and upper lines) and average IBF ± 6 Hz (external upper and lower lines). Rightmost inset: scatterplot labels/scales. (B) M1 power–time course. Modulation of M1 Power for each condition (active: colored continuous lines; sham: colored dotted lines) as a function of time. Time 0 represents the onset of the TMS burst. Please note the general increase in power for the active condition, relative to the sham condition when TMS is delivered at IBF (red continuous line). Effects of stimulation frequency and condition are presented in the left inset. The IBF conditions have been contrasted against the surrounding frequencies to directly test for the hypothesis that IBF TMS will preferentially entrain beta-oscillations compared to the surrounding frequencies. This is confirmed for active stimulation (*T9 = 2.92; *p* = 0.017; Cohen’s *d* = 0.68), while sham stimulation shows no difference between stimulation frequencies. (C) EMG power–time frequency analysis. Entrainment effects for EMG power as a function of frequency of stimulation and condition (as in Fig. 2A). (D) EMG power–time course. Modulation of EMG Power for each condition (active: colored continuous lines; sham: colored dotted lines) as a function of time. Please note the weaker and nonsignificant effects of EMG power (left inset).

**Fig. 3:**
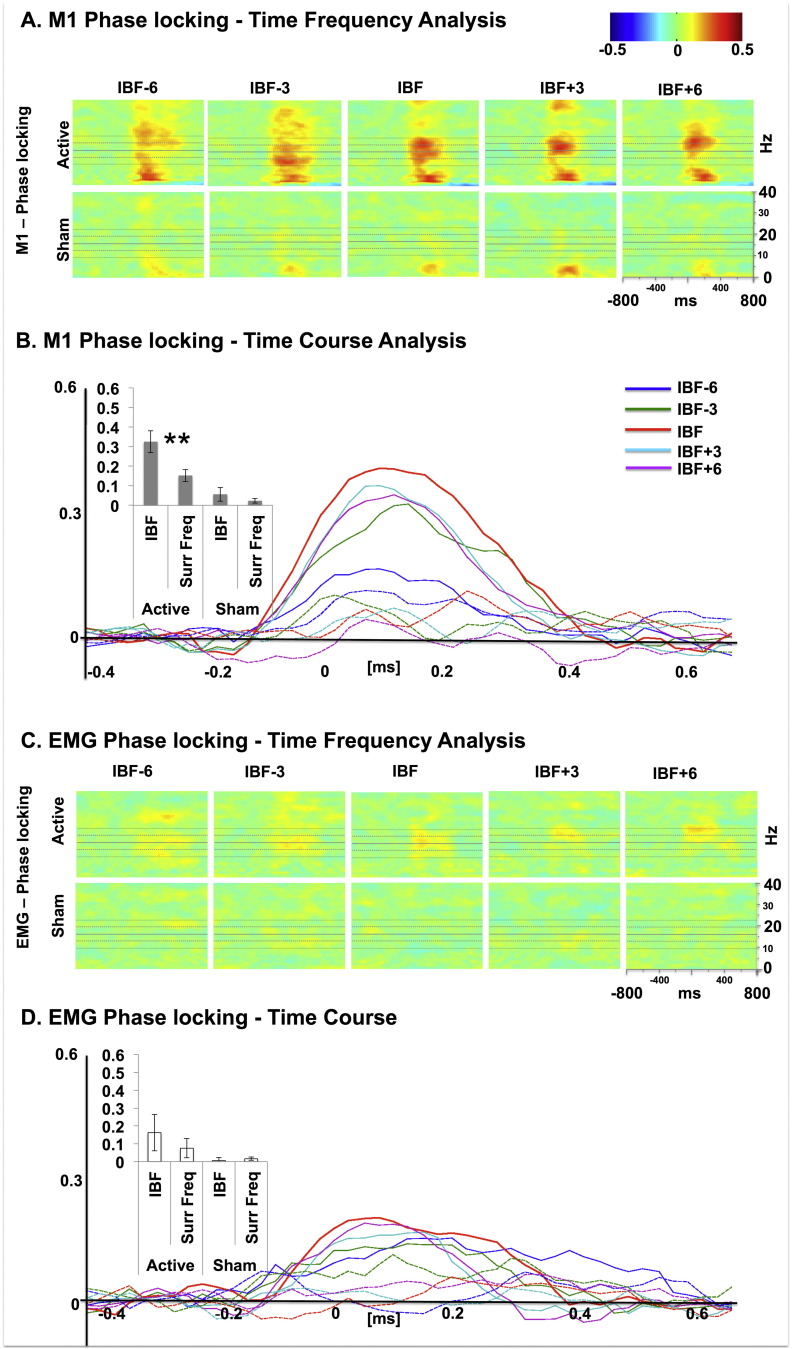
(A) M1 PLV–time frequency analysis. Entrainment effects for M1 phase locking value (PLV) as a function of frequency of stimulation (individual beta-frequency [IBF], central plot, and surrounding frequencies: IBF ± 3 Hz and IBF ± 6 Hz) and condition (active stimulation: upper row; sham stimulation: lower raw). The set of horizontal lines in scatterplots represent the average IBF (central line), average IBF ± 3 Hz (middle upper and lower lines), and average IBF ± 6 Hz (external upper and lower lines). Rightmost inset: scatterplot labels/scales. (B) M1 PLV–time course. Modulation of M1 PLV for each condition (active: colored continuous lines; sham: colored dotted lines) as a function of time. Time 0 represents the onset of the TMS burst. Please note the general increase in PLV for the active condition, relative to the sham condition when TMS is delivered at IBF (red continuous line). Effects of stimulation frequency and condition are presented in the left inset. The IBF conditions have been contrasted against the surrounding frequencies to directly test for the hypothesis that IBF TMS will preferentially entrain beta-oscillations compared to the surrounding frequencies. This is confirmed for active stimulation (**t(9) = 3.86; *p* = 0.004; Cohen’s *d* = 1.28), while sham stimulation shows no difference between stimulation frequencies. (C) EMG PLV–time frequency analysis. Entrainment effects for EMG PLV as a function of frequency of stimulation and condition (as in Fig. 3A). (D) EMG PLV–time course. Modulation of EMG PLV for each condition (active: colored continuous lines; sham: colored dotted lines) as a function of time. Please note the weaker and nonsignificant effects of EMG PLV (left inset).

**Fig. 4:**
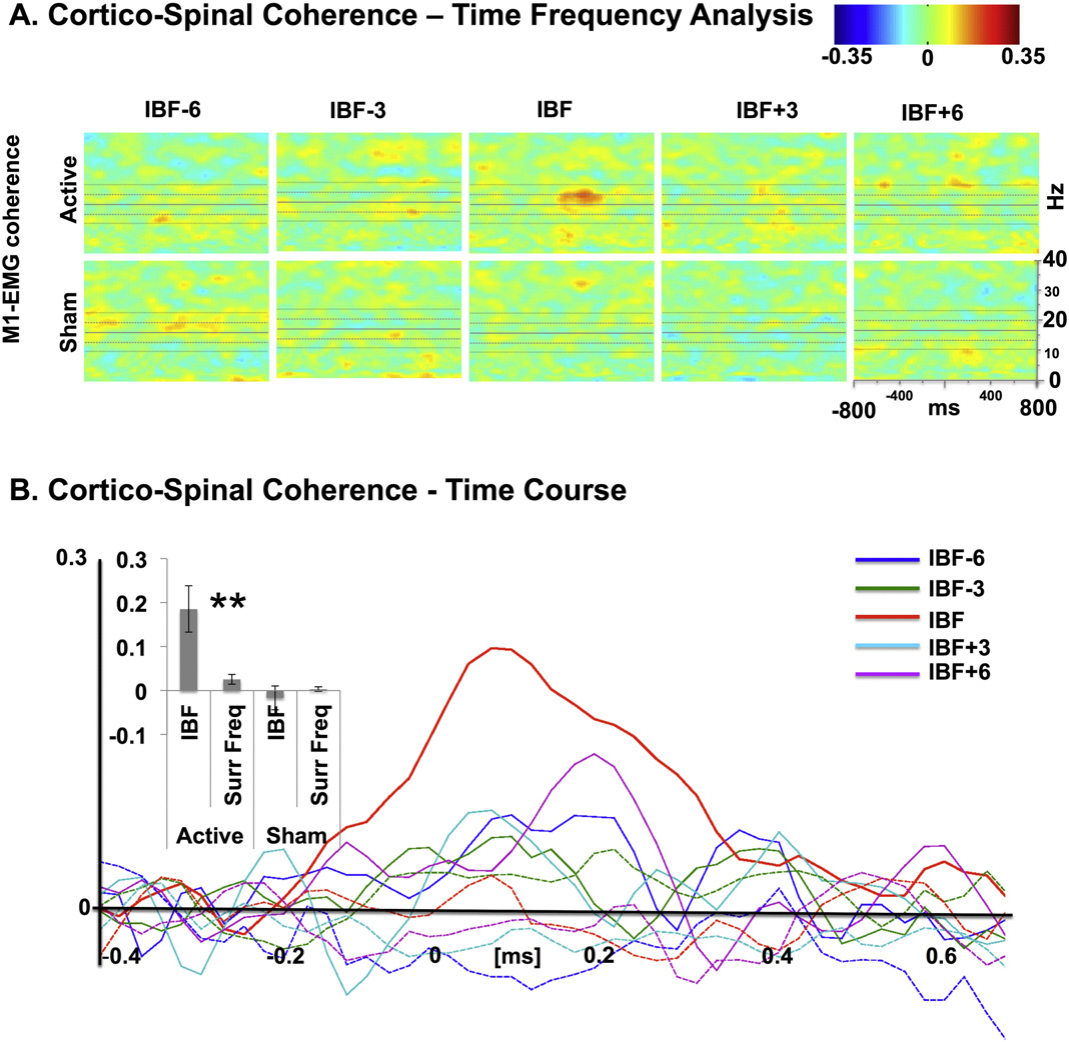
(A) Cortico-spinal coherence–time frequency analysis. Entrainment effects for cortico-spinal coherence as a function of frequency of stimulation (individual beta-frequency [IBF], central plot and surrounding frequencies: IBF ± 3 Hz and IBF ± 6 Hz) and condition (active stimulation: upper row; sham stimulation: lower raw). The set of horizontal lines in scatterplots represent the average IBF (central line), average IBF ± 3 Hz (middle lower and upper lines), and average IBF ± 6 Hz (external upper and lower lines). Rightmost inset: scatterplot labels/scales. (B) Cortico-spinal coherence–time course. Modulation of cortico-spinal coherence for each condition (active: colored continuous lines; sham: colored dotted lines) as a function of time. Time 0 represents the onset of the TMS burst. Please note the general increase in cortico-spinal coherence for the active condition, relative to the sham condition, when TMS is delivered at IBF (red continuous line). Effects of stimulation frequency and condition are presented in the left inset. The IBF conditions have been contrasted against the surrounding frequencies to directly test for the hypothesis that IBF TMS will preferentially enhance cortico-spinal coherence compared to the surrounding frequencies. This is confirmed for active stimulation (**t = 3.66; *p* = 0.006; Cohen’s *d* = 1.48), while sham stimulation shows no difference between IBF and other stimulation frequencies.

Comparing the strength of the rhythmically entrained brain activity induced by the TMS pulse over motor cortex, we found maximum entrainment effects for rhythmic TMS set at the IBF. This is shown by the power spectra and time course of power at the stimulated frequencies (see Fig. 2A–C) and reflected in maximum beta-power boosting observed in a narrow beta-band centered around the stimulation frequency of the IBF. TMS-induced beta-power during the stimulation train, as identified in the estimated sensorimotor virtual channel (see methods for details), was significantly weaker for the surrounding frequencies. This was tested using a repeated-measures 2 × 2 ANOVA with factors stimulation frequency (IBF vs. surrounding frequencies) and TMS condition (active vs. sham). This design has the advantage that any differences between frequencies may not be due to confounding factors, such as monotonous effects of frequency, and crucially, an interaction between TMS condition and stimulation frequency can therefore only be attributed to a resonance effect on brain activity. The ANOVA revealed a main effect of “TMS condition” (“active” vs “sham”) [*F*(1,9) = 8.57; *p* = 0.017; *η*^2^ = 0.49)], a main effect of “stimulation frequency” (IBF vs surrounding frequencies) [*F*(4,36) = 7.74; *p* = 0.021; *η*^2^ = 0.46], and an interaction between condition and frequency [*F*(4,36) = 5.84; *p* = 0.039; *η*^2^ = 0.39] (see Fig. 2A). The latter indicates that stimulation at the individual beta-frequency compared to the surrounding frequencies was enhanced only for the active but not the sham stimulation condition. In order to assess the specific contributions of the active and the sham conditions to these effects, two separate one-way repeated-measures ANOVA with factor “stimulation frequency” were performed for active and sham conditions. Results confirmed that the active [*F*(1,9) = 8.51, *p* = 0.017; *η*^2^ = 0.49] but not the sham condition [*F*(1,9) = 0.99, *p* = 0.34; *η*^2^ = 0.10] selectively enhanced motor beta-power at IBF relative to the surrounding frequencies. Therefore, as can be appreciated by simple visual inspection (see Fig. 2, Fig. 3, Fig. 4) as well as by statistical tests, none of the other frequencies show higher values compared to the IBF, nor any response to surrounding frequency stimulation are found to be distinctive from the other surrounding frequencies. We further tested whether there might be a graded effect of the distance of the stimulation frequency from the peak frequency; a paired *t*-test between the averages of IBF ± 6 and IBF ± 3 Hz did not reveal any significance [*t*(9) = − 0.615, *p* > 0.5]. Likewise, there were no significant differences between the averages of IBF − 6 and IBF − 3 Hz on the one hand, and IBF + 3 and IBF + 6 Hz on the other hand [*t*(9) = 0.178, *p* > 0.5]. This therefore rules out that the resonance effect described above may have been due to a more trivial (e.g., linear monotonous) effect of frequency but also does not support our third hypothesis that there should be a graded entrainment effect as a function of frequency (see discussion for details).

### 3.2 TMS effects on EMG beta-power

We furthermore investigated whether our TMS manipulation affected the EMG signal, even though individual TMS pulses were delivered at subthreshold intensities. Therefore, we ran a 2-way repeated-measures ANOVA with factors “stimulation frequency” and “TMS condition” on the EMG signal, which showed no significant main effects of condition or frequency nor interactions between these two factors (*F*(1,8) = 4.42; *p* = 0.07; *η*^2^ = 0.35), although revealing a slight trend in the same direction as for sensorimotor beta-power (Fig. 2D–F).

This result suggests that the emergence of a spectral pattern in the EMG activity was weak but that the entrainment of motor cortical activity progressed downstream to the spinal cord. Further analysis of the EMG signal on single trials for each participant excluded TMS-evoked motor potentials as the origin of these marginally significant trends (see below), but we note that we cannot exclude any spinal effects that may have not become detectable with our surface EMG recordings.

### 3.3 EMG inspection for presence/absence of motor-evoked potentials (MEPs)

To investigate whether the trend of enhanced beta-band activity for the IBF frequency might have been caused by suprathreshold EMG activations – or reflected a more subtle process without eliciting a full-scale MEP – we analyzed EMG traces for the occurrence of such MEPs. We also investigated a potential increase in total EMG power (rather than investigating frequency-specific changes over the entire TMS train) following each individual TMS pulse. The repeated-measures 2-way ANOVA for the total EMG variance revealed no significant main effects of condition or frequency (condition sham vs TMS: *F*(1,8) = 0.93, *p* > 0.3; frequency: *F*(1,8) = 0.31, *p* > 0.87), nor in the frequency of “detected MEP's” (even using a rather liberal criterion for MEP detection, see above; condition sham vs TMS: *F*(1,8) = 0.49, *p* > 0.5; frequency: *F* = 0.72, *p* > 0.5).

### 3.4 TMS effects on cortical phase locking

A key prediction for the entrainment of brain activity through rhythmical TMS bursts at the IBF is that this should result in phase alignment of brain activity to the externally imposed rhythm. We therefore calculated the phase-locking value (PLV) between the TMS pulses and the EEG signal recorded from sensorimotor cortex. Accordingly, the 2-way ANOVA with the factors stimulation frequency (IBF vs. surrounding frequencies) and TMS condition (active vs. sham) performed on sensorimotor beta phase locking revealed a main effect of condition [*F*(1,9) = 28.55; *p* < 0.001; *η*^2^ = 0.76)], a main effect of stimulation frequency [*F*(1,9) = 13.36, *p* = 0.005; *η*^2^ = 0.59], and an interaction between these two factors [*F*(1,9) = 6.85; *p* = 0.028; *η*^2^ = 0.43] (see Fig. 3A), suggesting that rhythmical brain stimulation via TMS in the beta-band enhances phase locking in the beta range differently for active and sham conditions. Critically, this effect depends on the specific frequency of stimulation, it being stronger for the individual beta-frequency compared to the surrounding frequencies. The two one-way ANOVAs separately performed for the active and the sham condition confirm that the active [*F*(1,9) = 14.91, *p* = 0.004; *η*^2^ = 0.62] but not sham beta stimulation [*F*(1,9) = 1.08; *p* = 0.33; *η*^2^ = 0.11] significantly enhance phase locking values at IBF vs. surrounding frequencies.

### 3.5 TMS effects on EMG beta phase locking

The same 2-way ANOVAs conducted on the EMG signal for phase locking revealed no significant main effects nor interactions between Condition and Frequency [*F*(1,8) = 3.12; *p* = 0.11; *η*^2^ = 0.28] (Fig. 3B).

### 3.6 Individual beta-frequency gates cortical influences on spinal motor activity

Several studies provide evidence that beta-oscillations have a modulatory impact on motor control, indexed via cortico-spinal signal interactions (e.g., Schoffelen et al., 2005, Hari and Salenius, 1999, Mima and Hallett, 1999). If this were the case, then entrainment of sensorimotor beta through subthreshold stimulation should propagate to spinal levels, even when no active motor task is performed. We therefore looked at how entrainment of sensorimotor beta at individual and surrounding frequencies influences cortico-spinal coherence.

We found that only rhythmic TMS at the IBF resulted in significant cortico-spinal coupling [Fig. 4]. The 2-way repeated-measures ANOVA with the factors stimulation frequency (IBF vs. surrounding frequencies) and TMS condition (active vs. sham) showed a main effect of “TMS condition” [*F*(1,8) = 15.95, *p* = 0.004; *η*^2^ = 0.67] and “stimulation frequency” [*F*(1,8) = 7.52, *p* = 0.025; *η*^2^ = 0.48], and a significant interaction between these two factors [*F*(1,8) = 12.60; *p* = 0.0075; *η*^2^ = 0.61]. The one-way ANOVAs performed separately for each condition confirmed that the frequency-specific increase in cortico-spinal coherence occurred only in the active TMS condition [*F*(1,8) = 13.43, *p* = 0.006; *η*^2^ = 0.63] but not for the sham conditions [*F*(1,8) = 0.58, *p* = 0.47 *η*^2^ = 0.07]. Therefore, TMS stimulation delivered at sub-motor-threshold intensities entails cortico-spinal coherence when delivered at the resonance frequency (Fig. 4).

## 4 Discussion

In this report, we have shown that rhythmic TMS over motor cortex at the individual peak frequency of intrinsic beta-oscillations causes stronger oscillatory synchronization compared to other nearby frequencies. Furthermore, rhythmic subthreshold stimulation at individual beta-band frequencies caused increased cortico-spinal coherence. Our results reveal an inherent physiological property of cortical circuits in that the (individually specific) inherent rhythms of these circuits determine the degree of signal propagation within the network even at rest. These results support accounts that propose an important role for oscillatory mechanisms for signal propagation through nervous systems (Salinas and Sejnowski, 2002, Akam and Kullmann, 2010) and provide causal evidence that individual motor beta-frequency oscillations specifically mediate cortico-spinal signal interactions (cf. Schoffelen et al., 2005, Hari and Salenius, 1999, Mima and Hallett, 1999).

### 4.1 Rhythmic TMS induces cortical but not muscular oscillatory beta entrainment

Comparing the effects of the rhythmic TMS conditions over the motor cortex on brain activity, we found clear entrainment effect with maximum impact for stimulation at IBF on a number of oscillatory parameters as shown for example by the power spectra and time course of power and phase locking values at the stimulated frequencies analysis of EEG during rhythmic TMS. However, the same type of analyses performed on the EMG signal did not reveal any significant modulation by the rhythmic protocol, independently of the frequency of stimulation.

The lack of a significant entrainment effect in beta-power or phase over the EMG signal of the contralateral target muscle suggests that the rhythmic stimulation did not elicit significant, induced activity in the peripheral target muscle. These results indicate that the sensorimotor beta entrainment observed here is predominantly of cortical origin and unlikely to be caused by sensory-motor feedback induced by peripheral muscle activation (Di Lazzaro et al., 1999). However, we note that from this we cannot exclude the possibility of spinal effects that may not have elicited measurable effects in our target muscles.

### 4.2 Cortico-spinal connectivity is gated by individual beta peak frequency

We furthermore demonstrated that frequency-tuned stimulation of sensorimotor cortex maximally entrains oscillatory activity when the stimulation frequency matches the natural individual beta-frequency. We have also seen that the subthreshold TMS protocol had only relatively little direct impact at the spinal level per se, confirming that the entrained beta oscillation effects reported here occurred predominantly at the cortical level. This raised the question as to whether this entrainment had any impact on signal propagation to downstream spinal motor neurons. Beta-oscillations may be the consequence of the interactions between different network nodes within the motor system. We therefore asked whether any change in functional connectivity between sensorimotor cortex and spinal cord occurred during or following rhythmic subthreshold TMS. To this end, we computed cortico-muscular coherence between the cortical EEG signals obtained over sensorimotor cortex and the EMG recorded from the contralateral hand.

Indeed a surprising result of the present study is the specificity of the increase in cortico-spinal coherence in the beta-band when applying subthreshold TMS. This increased coherence was strongest when stimulation was applied at the intrinsic and individual beta-frequency. This cortico-spinal coherence is not a local phenomenon of the directly stimulated sensorimotor cortex but reflects a network effect. The activation of the motor system at its resonant frequency at rest thus had a significant impact on the efficacy of signal propagation – even in the absence of strong oscillations and at intensities that would otherwise not lead to such activation. Our results thus support theoretical accounts suggesting that oscillatory mechanisms can facilitate signal processing (Salinas and Sejnowski, 2002) and that conceptualize cortical circuits as band-pass filters (Akam and Kullmann, 2010). More generally, our results provide causal evidence for the physiological importance of individual peak frequency for neural information processing.

Finally, the observed increase in rhythmic patterning occurred in the absence of suprathreshold muscular activation. The EEG responses measured from scalp electrodes are thought to predominantly reflect local-field potentials (and therefore the sum of excitatory and inhibitory post-synaptic potentials, Nunez, 2000). By contrast, EMG signals recorded with surface electrodes placed over the muscle belly result from the summation of motor neuron action potentials arriving at the motor end plate.

How then can such subthreshold stimulation increase cortico-muscular coherence at rest? First, even when maintaining rest there may be a low-level tonic firing in some motor units, which results from spontaneous spiking in spinal motor neurons (Blankenship and Kuno, 1968). The increased cortico-spinal beta-coherence and the trend for increased beta-power could then results from increased temporal structuring at beta-frequencies of this spontaneous spiking activity. Second, there might be increased spiking activity following the TMS pulses but this may not be of sufficient strength to elicit a full-blown MEP that presumably reflects the synchronized activity of numerous fibers.

However, we note that coherence is a measure that is highly susceptible to effects of either volume conduction or common noise in the signals under consideration, and is relatively independent of the amplitude of such noise sources. Therefore, despite the distinctive frequency and condition specificity we observe here (and the implausibility of this being caused by any artifact), we sought further confirmation that these results were not confounded by the specific treatment of the EEG or EMG data.

**Fig. 5:**
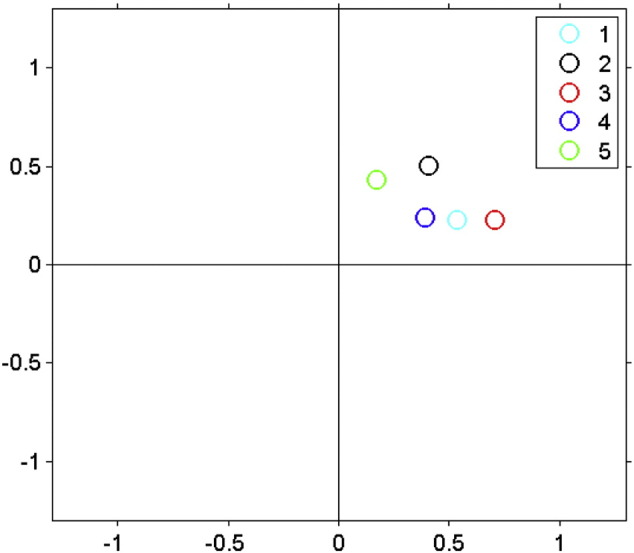
Complex cortico-spinal coherence for TMS: the complex valued coherence values show that all data points are off the real axis, indicating that the enhanced coherence for the IBF frequency (condition 3, in red) is not due to volume conduction or artifact removal. In the upper right inset: condition 1: IBF − 6 Hz; condition 2: IBF − 3 Hz; condition 3: IBF; condition 4: IBF + 3 Hz; condition 5: IBF + 6 Hz.

Any spurious or artifactual influences on the cortico-spinal coherence data reported here should occur with zero-phase lag, given that EMG and scalp electrodes were recorded simultaneously and our interpolation occurred on the very same samples in scalp channels and for the EMG. While physiological oscillations can indeed be relatively precisely phase synchronized (which would result in a near zero-phase-lag of the complex coherence-estimates), any deviation from the real axis of the complex coherence coefficients can only be explained by a non-instantaneous common signal and therefore would reflect a signal of physiological origin. We therefore determined the phase of the cortico-muscular coherence enhancement observed here. As shown in Fig. 5, all complex coherence values were indeed off the real axis. While the absolute magnitude of coherence was largest for the individual beta-frequency, the phase angle was smallest, indicating more synchronous phase alignment of the beta-oscillations in spinal cord and sensorimotor cortex. This frequency specificity of the effect and the non-zero-phase angle suggests that the cortico-spinal coherence effect was of physiological nature. We cannot safely conclude whether the effects we measured were directly generated in the motor cortex itself, or reflect an emergent property of the cortico-spinal loop responding to the enhanced rhythmic drive from motor cortex at its intrinsic rhythm. However, since we only stimulated the motor cortex, it is evident that an input at the system’s characteristic frequency enhances signal propagation within the cortico-spinal system as a whole.

In this respect, several studies have provided direct support for the cortico-muscular coherence in the beta-band reflecting a cortical drive (Schoffelen et al., 2005, Mima et al., 2000, Mima et al., 2001).

### 4.3 Additional note on spectral analysis during rTMS

Can our data set be explained by residual artifacts? While the TMS artifact here was of very short duration, the data immediately following the pulse were treated with an interpolation routine which unavoidably introduces some discontinuity that will appear in the Fourier transform with a peak at that particular stimulation frequency (i.e., the one under consideration). However, crucially, the artifact treatment was exactly the same under all conditions—sham and active TMS (cf. Thut et al., 2011a) as well as for all frequencies. Hence, any non-monotonous frequency- and/condition-specific effects cannot be explained by any discontinuities introduced by this data treatment. Any non-physiological frequency-specific effect should manifest itself in a monotonous dependency on frequency (considering the constant length of the artifact removal after each pulse and the monotonous variation of the inter-pulse-interval). The crucial 2 × 2 design comparing TMS at the IBF versus the surrounding peaks of both higher and lower frequency (IBF ± 3 and IBF ± 6 Hz) excludes such effects to contaminate the statistical inference on the effect of IBF stimulation versus non-IBF stimulation. Finally, despite the non-stationarity of these data, the key aspect here is the fact that the causal manipulation of driving the system at its intrinsic frequency leads to stronger effects in the motor system, proving the relevance of the innate rhythmicity for signal propagation in the motor system.

### 4.4 Conservative bias and the absence of graded entrainment effects

We note that one of our initial hypotheses was that there would be a graded effect of the distance of stimulation frequency from IBF, besides the discrete resonance effect (IBF vs satellites). The data collected in this study failed to show any such effect. This could be due to a number of reasons: (1) the frequencies chosen here were spaced relatively far away from the IBF, (2) the entrainment cycle here was rather short (5 pulses only) (see Hanslmayr et al., 2014 for effects of longer entrainment cycles) and of relatively low intensity, (3) our TMS equipment did not have the very latest option of delaying the recharge after a series of pulses causing somewhat enhanced noise levels, and (4) the artifact reduction techniques used in this study may be on the conservative side, potentially attenuating also cranial sources. Future studies should investigate these issues specifically, e.g., by choosing the distance of satellite frequencies individually for each participant as multiples of the individual width of the spectral peak. The current design was also conducted under considerable ethical constraints, implying each session to run for approximately 4–5 h given the limitation of the number of pulses and the rest periods in-between.

### 4.5 TMS vs. somatosensory-induced entrainment

Can our data set be explained by the rhythmic sensations (on the skull itself) associated with the active (but not the sham) TMS rhythmic protocol? While it was possible to control for click related unspecific effects of TMS by using our sham protocol, this procedure controls less well for the tactile sensations produced on the scalp by the rhythmic stimulation. However, we consider this alternative explanation far less likely since these would not involve a cortico-spinal circuitry as measured through beta-band effects in the EMG on the right arm (and coherence to EEG), which was increased specifically for the individual beta-frequency band.

### 4.6 Conclusion

To conclude, this study provides direct evidence for the causal relevance of the specific frequency profile of cortical circuits for signal propagation. It therefore shows that the impact of rhythmic TMS stimulation depends on the systems individual transfer function. Future studies may provide more detailed and mechanistic insight how subthreshold rhythmic signals can propagate to spinal levels, but here we already provide causal evidence for the special role of intrinsic brain rhythms for signal propagation.

## Figures and Tables

**Supplemental Fig.S1:**
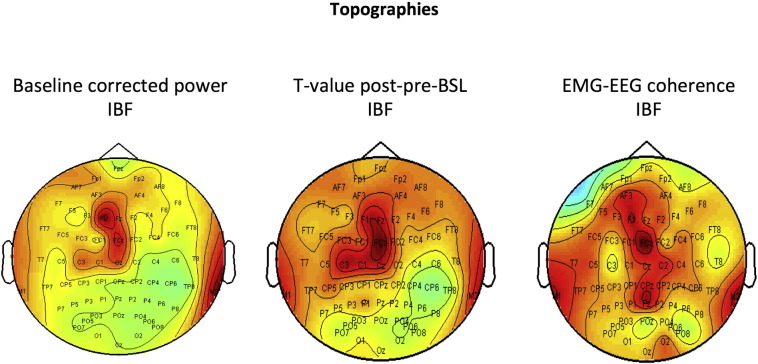
Sensor level topographies of TMS-induced effects.

**Supplemental Fig. S2:**
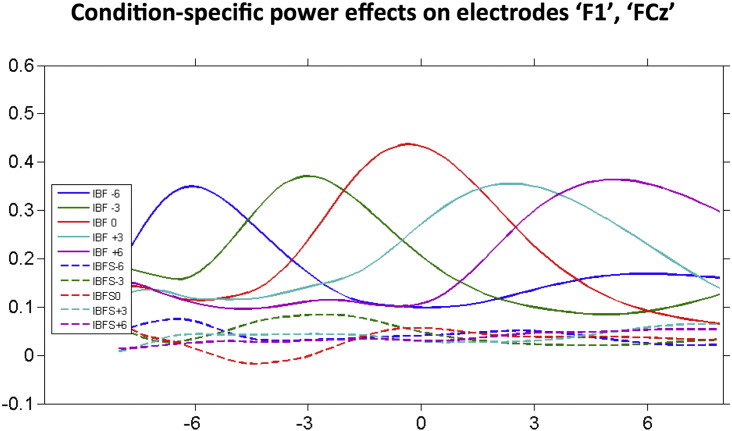
Power spectral density of TMS-induced effect aligned to the individual beta-frequency.

**Supplemental Fig. S3:**
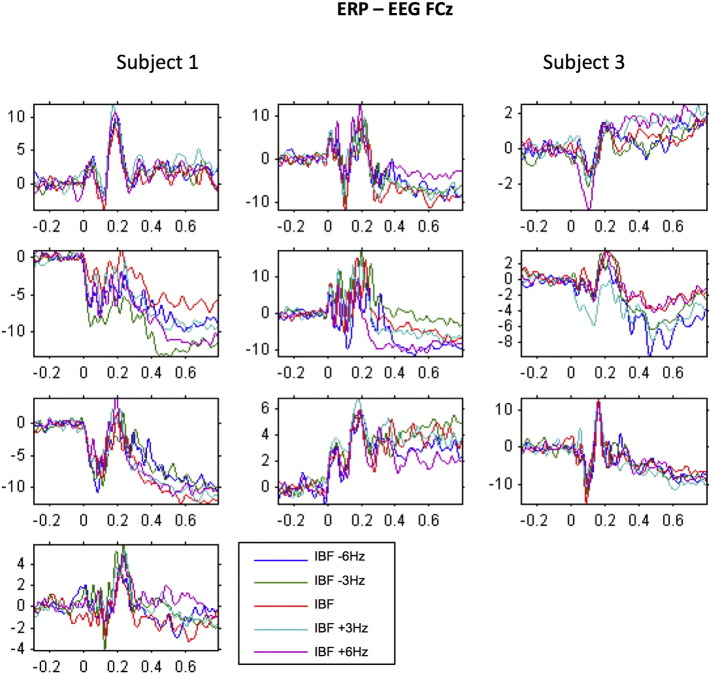
TMS-ERP in channel FCz.

**Supplemental Fig. S4:**
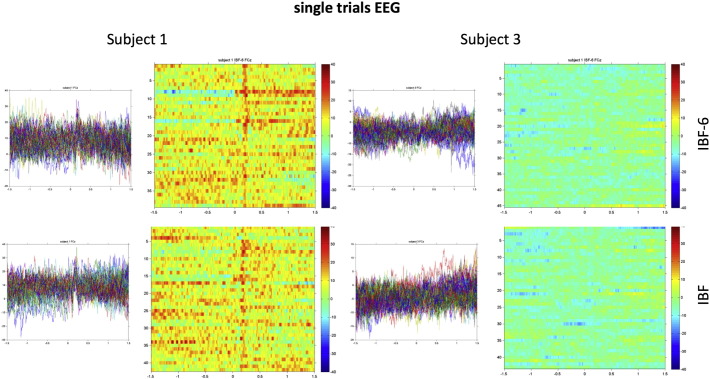
Single trial displays of EEG waveforms at FCz.

**Supplemental Fig. S5:**
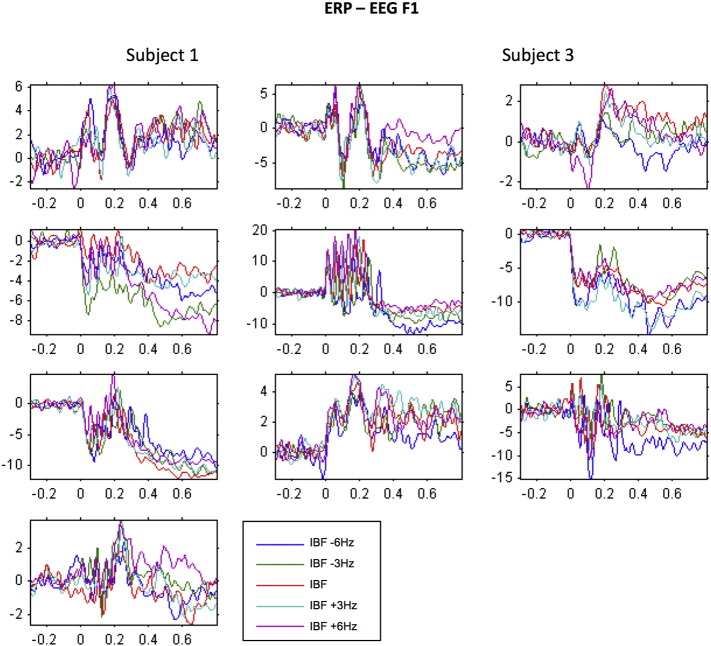
TMS-ERP in channel F1.

**Supplemental Fig. S6:**
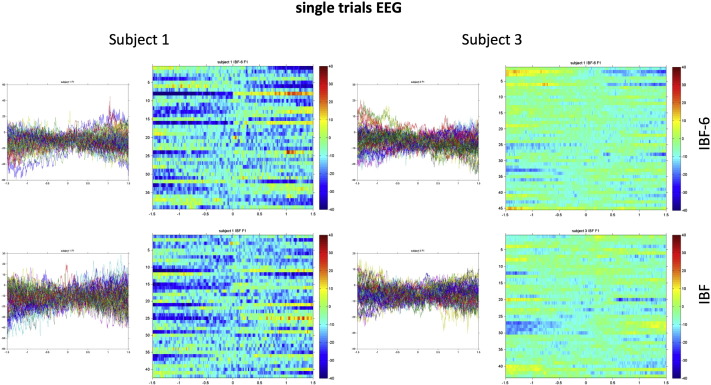
Single trial displays of EEG waveforms at F1.

**Supplemental Fig. S7:**
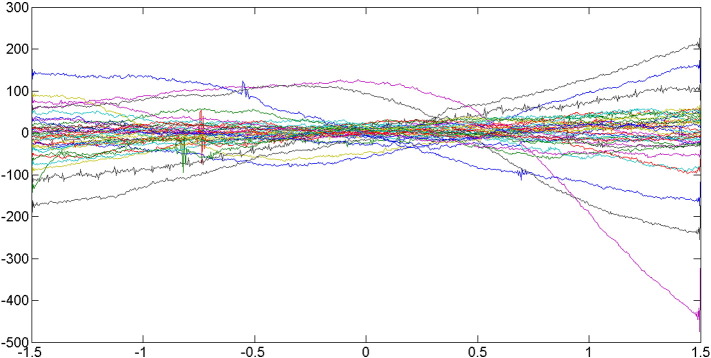
Single and average EMG traces of an exemplar participant.
